# Improved Monitoring and Assessment of Meteorological Drought Based on Multi-Source Fused Precipitation Data

**DOI:** 10.3390/ijerph19031542

**Published:** 2022-01-29

**Authors:** Si Chen, Qi Li, Wushuang Zhong, Run Wang, Dong Chen, Shihan Pan

**Affiliations:** 1 School of Resources and Environmental Science, Hubei University, Wuhan 430062, China; kathryncs123@hotmail.com (S.C.); 201931108010035@stu.hubu.edu.cn (Q.L.); Zhongwushuang@outlook.com (W.Z.); 2 Changjiang River Scientific Research Institute, Wuhan 430062, China; chendong@mail.crsri.cn; 3 Downstream Bureau of Changjiang Water Resources Commission, Wuhan 430062, China; 18086476966@163.com

**Keywords:** drought assessment, multi-source remote sensing, data fusion, spatio-temporal analysis

## Abstract

Meteorological drought, one of the most frequent climate-related disasters, causes great danger for human health and socioeconomic development. With an aim to improve the accuracy of meteorological drought monitoring, this study collected multi-source remotely-sensed precipitation products, i.e., the Tropical Rainfall Measuring Mission (TRMM), the Global Precipitation Measurement Mission (GPM), and Climate Hazards Group InfraRed Precipitation with Station data (CHIRPS), and compared their performance over Hubei Province, China. The geographic difference analysis was used to blend the best-fitted product with gauged precipitation data. Based on the fused dataset with verification, the spatio-temporal characteristics of drought were investigated. Results showed that GPM performed the best in precipitation numerical evaluation and event detection with a 5 mm/d threshold. The fused data accurately captured 80% of historical drought events and indicated that extreme annual droughts mainly occurred in the northern and northwestern regions, while slight, moderate, and severe droughts mainly occurred in the central and eastern parts. The short-term drought exhibited the highest frequency of 33% in summer and the lowest frequency of 27% in spring, while the medium-term drought showed a higher frequency in autumn and winter. This could be a preliminary assessment of drought based on multi-source fused precipitation data for precise drought outlook and risk management.

## 1. Introduction

The sixth assessment report of IPCC held on August 2021 indicates that climate change has affected the occurrence of a number of extreme weather and climate events in all regions of the world, including heavy rainfall, heat waves, and drought [1]. Increased drought risk under global warming has implications for at-risk individuals, communities and health systems [2]. In other words, drought can present perceived health and well-being effects through reduced water quantity, water quality, affected public health compromised hygiene and sanitation, food security, and air quality [3,4]. It was determined that 15 of the major droughts affected around 36.5 million people worldwide from 2003 to 2012 [2], and drought was responsible for the highest number of deaths worldwide between 1900 and 2019 (about 30%) [5]. From 1997 to 2001, one-half of the provinces in Iran experienced extensive and intensive drought events, resulting in more than a 10 billion USD loss in the agricultural sector [6]. Recent droughts have taken large economic and human tolls in the USA since the 1980s, with estimated losses exceeding 236 billion USD (CPI-adjusted) and 3865 deaths [7]. From January to October 2021, severe droughts affected 20.52 million people, 3448 hectares of crops, and caused direct economic losses of 20.14 billion in China [8]. It is urgent to carry out an accurate and timely assessment of drought under changing climate to reduce the aroused losses and risk. As one of the most important variables in drought monitoring, reliable precipitation input normally plays a crucial role in the hydrologic modeling and calculation of the most drought indices to quantify dry conditions [9]. It is vital to improve the precipitation data quality for precise drought assessment and outlook.

Two widely-used precipitation datasets are the measured data based on ground meteorological stations and remotely-sensed precipitation data with high spatio-temporal resolution based on satellite remote sensors. The measured rainfall data come from observation instruments such as rain gauges, raindrop spectrometers, ground-based radars, etc., which are able to be used as the reference basis for the accurate evaluation of precipitation authenticity inversion by remote sensing technology [10]. However, owing to the influence of the geographical environment and social-economic conditions, the distribution of ground meteorological stations is uneven, sparse, and even missing in areas with high altitudes and low population density [11]. Thus, it is difficult to capture the spatial distribution characteristics and intensity change of rainfall based on measured data. To overcome this disadvantage, the Global Precipitation Climatology Project (GPCP) [12] and the Climate Prediction Center (CPC) Merged Analysis of Precipitation (CMAP) products appeared in the 1970s, providing monthly precipitation data with a global scale [13]. In 1997, it ushered a new era of global precipitation remote sensing monitoring by carrying the world’s first satellite-borne precipitation radar, i.e., the Tropical Rainfall Measuring Mission (TRMM) [14]. Subsequently, TRMM Multi-satellite Precipitation Analysis (TMPA), Precipitation Estimation from Remotely Sensed Information using Artificial Neural Networks (PERSIANN), Climate Prediction Center Morphing (CMORPH), Global Precipitation Measurement Mission (GPM), and other products have emerged, providing abundant datasets for global precipitation. These widely-used remote sensing precipitation products can precisely capture the drought of contiguous regions in space with the advantages of broad coverage and high spatio-temporal resolution. However, certain systematic deviation and considerable spatial variability properties existed in the products due to the influence of terrain, altitude, latitude, precipitation intensity, and precipitation inversion methods [15].

It has been reported that the TRMM 3B42 product performs well in reproducing annual and monthly precipitation; in contrast, it performs poorly in precipitation frequency and intensity due to underestimating slight precipitation and overestimating the precipitation amount in mountainous and plain areas [16]. Wang et al. analyzed a number of representative results of TRMM satellite precipitation in Mainland China published in the Web of Science Core Collection database and Chinese core journal and found 55.5% of scholars believed that TRMM precipitation data exhibited overestimates, and 25.4% of scholars found underestimates over the high-altitude areas in China. Besides, 48.8% of the scholars believed that TRMM was consistent with observations in low altitude areas in China, while 39% of scholars believed it overestimated the real data [11]. Tan et al. demonstrated that TRMM products still had deviations due to the complexity of the geographical environment, sensor calibration, algorithm, and other reasons, and the accuracy verification was mostly concentrated in tropical regions [17].

NASA launched the Global Precipitation Measurement Mission (GPM) based on TRMM in 2014, which aimed to provide a new generation of quasi-global satellite remote sensing data products with higher accuracy and resolution. GPM products carrying the latest dual-band radar system improved the performance of the microwave radiometer compared to the generation of TRMM products [18], extending the spatial coverage to 60° S–60° N and spatial resolution to 0.1° [19]. Tang et al. conducted GPM and TRMM data as an example to analyze the accuracy of products from April to December 2014 in Mainland China over six typical constituencies and found that GPM had a better performance at both sub-daily and daily timescales over the mid-latitude and high-latitude areas [20]. Xu et al. analyzed the accuracy of GPM and TRMM products during the rainy season in 2014 based on the data of high-density meteorological stations in the Southern part of the Qinghai-Tibet Plateau and found that GPM tended to underestimate the number of slight rain events while the TRMM turned out to overestimate them [21]. Yang et al. stated that the accuracy of hourly GPM precipitation data should still be conducted for further verification at long-term series in the Shuaishui River Basin [22]. Consequently, the accuracy verification of GPM products is still inadequate due to their limited application period.

Climate Hazards Group InfraRed Precipitation with Station data (CHIRPS) precipitation products have a high spatial resolution (0.05°) and long time series ranging from 1981 to near-present, which has been widely applied in the study of drought in Brazil, Cyprus, Nepal, Italy, Mozambique, China, and other countries, and achieves satisfying application performance [23]. Gao et al. evaluated the effectiveness of drought monitoring in the Haihe River Basin of China using CHIRPS data and concluded it could be a valuable complement to gauged precipitation [24]. Similarly, Guo et al. suggested CHIRPS could properly capture the drought characteristics at various time scales with the best performance at a three-month time scale over the Lower Mekong River basin from January 1981 to July 2016 [25]. Zhong et al. verified the applicability of CHIRPS with comparison to other satellite precipitation products in China and concluded that CHIRPS showed better performance in matching the spatial pattern of typical drought events than the PERSIANN-Climate Data record (PERSIANN-CDR) [26].

Since the applicability of different satellite remote sensing products varies to different applied purposes and regions, existing studies have tested the data accuracy with remarkably discrepant spatial-temporal resolutions and scales. Guo et al. verified the applicability of TRMM 3B43 in the Huang-Huai-Hai Plain and revealed the seasonal spatio-temporal variation characteristics of precipitation in different years for this region [16]. Mohammad et al. compared the ability of precipitation products (i.e., TRMM, GPM, CHIRPS, and ECMWF Reanalysis v5 (ERA5)) to capture drought events in Iran and concluded that GPM and TRMM exhibited better monitoring performance while the results of CHIRPS showed the lowest accuracy [27]. Mohammad et al. evaluated the accuracy and applicability of the new satellite precipitation product SM2RAIN in Pakistan, which was verified to be feasible for most areas in Pakistan [28]. Using the data accuracy evaluation, the optimal products for the research area can be selected to monitor extreme rainfall, floods, and drought disasters, improving the ability to capture and forecast these extreme climatic events.

Considering the wide applicability of TRMM, GPM, and CHIRPS precipitation products, this paper conducts a precision evaluation to select the product with the best performance regarding data fusion processing for drought monitoring and assessment in Hubei Province. Then, the spatial and temporal distribution characteristics of drought in Hubei Province based on the fused dataset are analyzed. This study is expected to provide an evaluating method for the accuracy of multi-source remote sensing precipitation products and offer scientific guidance for accurate drought assessment based on a fused dataset in Hubei Province.

## 2. Materials and Methods

### 2.1. Study Area

**Figure 1:**
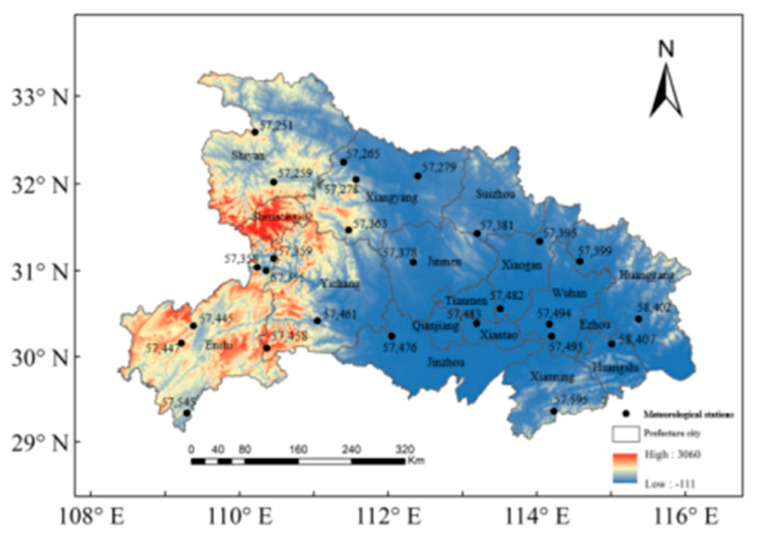
Distribution of meteorological stations and prefecture-level cities in Hubei Province.

Hubei province is located in the midland part of China (29–34° N and 108–117° E) as an important main grain-producing area and transportation hub (Figure 1). The meteorological gauged stations distributed in Hubei are listed in the following Table 1. It is a typical continental east coast type (monsoon type) climate zone, with annual precipitation ranging from 800 mm to 1600 mm per year, which is higher than the national average level. However, the distribution of precipitation time series is unevenly affected by monsoon, and the precipitation in the period from May to September approximately accounts for 60% of the annual rainfall. Especially affected by the plum-rain season, the rainfall from mid-June to mid-July lasts for the longest period and shows the highest intensity. Hubei province frequently suffered from natural disasters, with floods and drought emerging alternately, accompanied by hurricanes, freezing, and other meteorological disasters in the past 20 years. As recorded in the yearbook of meteorological disasters in China, enormous casualties and economic effects were caused by the severe droughts that occurred in the years 2005, 2007, 2010, 2013, and 2014 [29].

### 2.2. Data

Ground precipitation observations and satellite precipitation data covering Hubei province are collected for years 2002–2017. Twenty-six meteorological stations of the daily precipitation data in the study area derived from the National Meteorological Data Center (http://data.cma.cn/, accessed on 15 August 2021) are obtained. The satellite-based daily precipitation product TRMM 3B42V7 originates from the national aeronautics and space administration (NASA) data sharing website (http://trmm.gsfc.nasa.gov/ accessed on 15 August 2021), with the coverage of 50° S–50° N, 180° W–180° E and a spatial resolution of 0.25° × 0.25°. GPM_3IMERGDF daily precipitation products with the coverage of 50° S–50° N, 180° W–180° E and a spatial resolution of 0.1° × 0.1° are provided by the GPM team from the NASA sharing website (https://disc.gsfc.nasa.gov/ accessed on 15 August 2021). CHIRPS was developed by the United States Geological Survey (USGS) and the Climate Hazards Group at the University of California with multi-temporal resolution, which has been applied since 1981. For this study, the latest version of the CHIRPS daily precipitation products with the coverage of 50° S–50° N, 180° W–180° E and a 0.25° × 0.25° spatial resolution are obtained from the CHIRPS webpage (https://data.chc.ucsb.edu/products/CHIRPS-2.0/ accessed on 15 August 2021). The above precipitation datasets are all aggregated into a monthly scale for analysis in this study.

### 2.3. Method

#### 2.3.1. Precision Evaluation Index of Remote Sensing Precipitation Data

The correlation coefficient (CC), root mean square error (RMSE), mean error (ME), and relative bias (BIAS) were employed to evaluate the performance of TRMM, GPM, and CHIRPS precipitation data based on the observations as reference values. As shown in the following equations, the *CC* was used to quantify the degree of linear correlation between the validated and referenced series; the *RMSE* was used to evaluate the level of satellite product error and reflect the precision of precipitation products; the *ME* estimated the average error and helped to capture the bias of the satellite data in relation to the observations; the *BIAS* was used to compare the error level between the satellite products and the observations on the same scale, with dimension of percent. The ideal values for *CC*, *RMSE*, *ME*, and *BIAS* are 1, 0, 0, and 0, respectively.

(1) $$CC=\frac{\sum_{i=1}^{n}\left(G_{i}-\bar{G}\right)\left(P_{i}-\bar{P}\right)}{\sqrt{\sum_{i=1}^{n}{\left(G_{i}-\bar{G}\right)}_{}^{2}}\sqrt{\sum_{i=1}^{n}{\left(P_{i}-\bar{P}\right)}_{}^{2}}},$$

(2) $$RMSE=\sqrt{\frac{1}{n}\sum_{i=1}^{n}{\left(P_{i}-G_{i}\right)}_{}^{2}},$$

(3) $$ME=\frac{1}{n}\sum_{i=1}^{n}\left(P_{i}-G_{i}\right),$$

(4) $$BIAS=\frac{\sum_{i=1}^{n}\left(P_{i}-G_{i}\right)}{\sum_{i=1}^{n}G_{i}},$$

where $G_{i}$ and $\bar{G}$ are the observed precipitation in month *i* and the overall monthly average observations, respectively. *Pi* and $\bar{P}$ are the remotely-sensed precipitation in month *i* and overall monthly average values, respectively. The probability of detection (*POD*) and false alarm ratio (*FAR*) were used to evaluate the precision of capturing precipitation events. As shown in the following equations, the *POD* reflected the accuracy of satellite remote sensing products in rainy event detection while the *FAR* represented the false rate. The ideal values for *POD* and *FAR* are 1 and 0, respectively.

(5) $$POD=\frac{A}{A+B}$$

(6) $$FAR=\frac{C}{A+C}$$

where *A* is the number of rainy events that are both detected by the remote sensing dataset and observations, *B* is the number of rainy events that are detected by the observations but not the remote sensing dataset, *C* is the number of rainy events that are detected by the remote sensing dataset but not the observations.

#### 2.3.2. Precipitation Data Fusion Processing

Satellite precipitation products provide high-resolution and spatially continuous precipitation. However, the precipitation estimates often show large systematic bias and random error in drought monitoring due to the occlusion by clouds, inadequate and time-discontinuous sampling, as well as imperfections in retrieval algorithms [30]. Hence, satellite precipitation data can be blended with ground gauged observations which are spatially sparse but quite accurate to greatly improve the accuracy and enhance its potential applicability [31]. Currently, the representative methods for precipitation data fusion processing include ordinary Kriging, conditional merging, Kriging with external drift, and bias correction [32,33,34,35]. For this study, the Geographical Difference Analysis (GDA) method, which is simple and practical, is used to perform the data fusion processing for the observed and remote sensed data [36,37]. The procedure is as follows:

1. Calculating the differences between the observed precipitation data and the satellite data for the 26 meteorological stations;
2. Applying the method of Inverse Distance Weight (IDW) [37] to interpolate the differences in the grid cell of the satellite product;
3. Adding the interpolated differences onto the satellite data. Negative values can be generated, which are simply set to 0 [9].

#### 2.3.3. Drought Monitoring Based on Standardized Precipitation Index (SPI)

The SPI [38] is used widely for drought monitoring and comparative drought assessment at different temporal and spatial scales [39]. In addition, the calculation of SPI is quite easy, with precipitation data serving as the only input data. Since SPI does not consider the influence of soil moisture, evapotranspiration, and other factors, it can fully display the effect of precipitation fusion data in this paper. When computing the SPI series, the historical precipitation data of the gauged stations are firstly fitted to a gamma distribution. Then the SPI value is the standard normal quantile with the non-exceedance probability calculated as the accumulated probability of the observed precipitation amount from the fitted gamma distribution. Hence, the SPI is a z-score and represents a departure from the mean in standard deviation units [40].

The SPI with 3-month, 9-month, and 12-month time scales (i.e., SPI-3, SPI-9 and SPI-12) were conducted to analyze the characteristics of short, medium, and long-term drought in this study. The SPI values were developed by the National Drought Mitigation Center (https://drought.unl.edu/Monitoring/SPI/SPIProgram.aspx accessed on 18 September 2021). According to the classification standard for meteorological drought from China Meteorological Administration, different drought categories can be classified based on the SPI values, as shown in Table 2.

## 3. Results

### 3.1. Precision Evaluation of Satellite Precipitation Products

**Figure 2:**
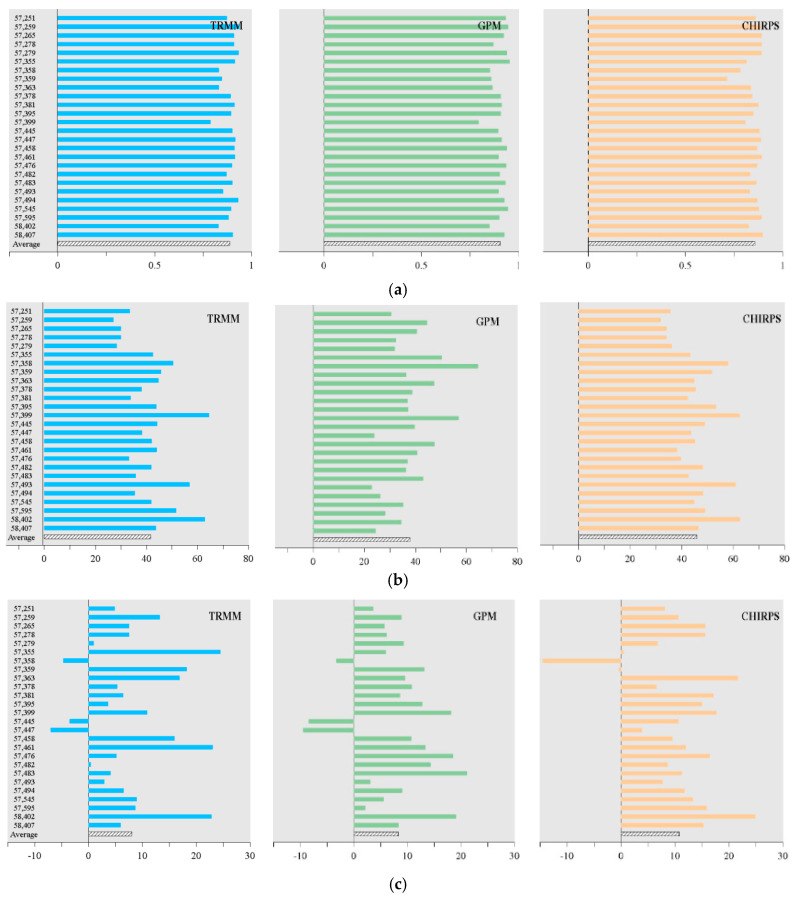

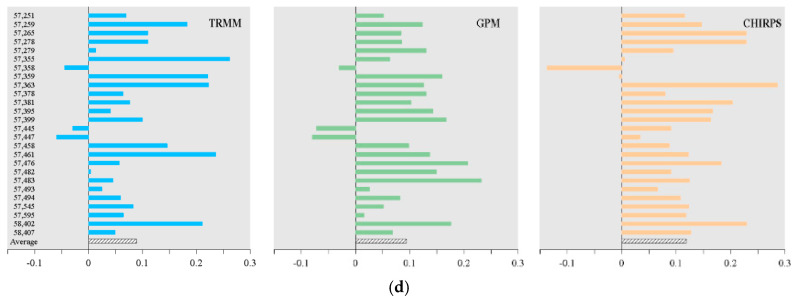
Precision evaluation criteria of TRMM, CHIRPS and GPM based on 26 meteorological stations ((**a**) *CC*, (**b**) *RMSE*, (**c**) *ME*, and (**d**) *BIAS*).

The *CC*, *RMSE*, *ME*, and *BIAS* values between the satellite precipitation data and observations at monthly scale from 2002–2007 over Hubei province were plotted in Figure 2. The average values of the 26 stations for each criterion were presented at the bottom of the figure. From Figure 2a, it can be seen that most stations for all three satellite products exhibited *CC* values larger than 0.8, which indicated that the satellite datasets all showed a strong correlation with observations. Figure 2b show that the station mean *RMSE* values of TRMM, GPM, and CHIRPS were 41.68, 37.95, and 45.85, respectively, and the GPM had the lowest *RMSE* values among all products. Meanwhile, the *RMSE* values showed rather large fluctuations among the stations, indicating that the overall error level of the precipitation product had a large difference at various stations. Figure 2c,d present the mean values of *ME* and *BIAS* of TRMM, GPM, and CHIRPS products to be 8.02, 8.31, and 10.81, respectively, and 8.9%, 9.3% and 11.8%, respectively. In terms of *ME* and *BIAS*, the results showed that the TRMM and GPM exhibited better performance than CHIRPS. Moreover, except for several stations, e.g., stations 57,358, 57,445, and 57,447, the rest of the stations all exhibited overestimates by the satellite products, which may be related to the complex terrain and climate of the western area in Hubei Province [41], and raindrops were affected by wind around these stations and thus hindered the observation of precipitation [42]. Overall, the performance of GPM and TRMM were comparable at most stations with low *RMSE*, *ME*, and *BIAS* and high *CC* values, which were better than that of CHIRPS products.

Table 3 present the *POD* and *FAR* values of the satellite products at 0.5 mm/d and 5 mm/d precipitation thresholds widely used to represent different rainfall intensities, which were performed to detect the rainy and heavy rainy months considering the average actual rainfall in Hubei province, respectively [43,44]. It was shown that three satellite products had better performance in detecting rainy rather than heavy rain months, and GPM showed the best performance for detecting heavy rain months compared to other satellite products with the largest *POD* and the lowest *FAR* values.

Specifically, the mean *POD* values of the three products were 0.947, 0.996, and 0.997 at the threshold value of 0.5 mm/d, respectively, almost all stations of the three products reached 0.9 or above and the values of CHIRPS and GPM reached 1 at most stations. The mean *FAR* values were 0.040, 0.048, and 0.079, respectively, less than 0.1 at each station, indicating the three satellite products had good performance to capture the occurrence of rainfall events considering the *POD* and *FAR*. Compared with 0.5 mm/d, the accuracy of capturing heavy rain for all products decreased, and the mean *POD* values were 0.827, 0.844, and 0.783, respectively. The *POD* values of three satellite products of all stations reached 0.8 or above at most stations, but the values were low for several stations. For example, the values of CHIRPS were 0.49 and 0.43 for Station 47,358 and 57,359, and the values of TRMM were 0.55 for Station 57,251, while no such poor result occurred with GPM. The mean values of *FAR* were 0.325, 0.295, and 0.356 for TRMM, GPM, and CHIRPS, respectively, and the *FAR* values at all stations for the three products were around 0.3. However, there existed an abnormal situation where TRMM showed *FAR* values of 0.50 and 0.53 for Station 57,359 and 57,363, and no stations of the GPM and CHIRPS showed values higher than 0.5.

Based on the results of the above indices, TRMM and GPM showed better performance than CHIRPS for precipitation numerical analysis and event detection. In terms of TRMM and GPM, GPM was significantly outperformed over TRMM for most stations in Hubei Province, even though GPM exhibited almost equal accuracy with TRMM for the *ME*, *BIAS* values, and 0.5 mm/d threshold in capturing precipitation events. Therefore, it can be considered that the GPM precipitation product was the optimal dataset with satisfying performance in drought monitoring in Hubei Province, and it was selected to carry out the following analysis.

### 3.2. Validation of Typical Drought Events

Blending GPM data and ground gauged observationsnusing a precipitation data fusion method formed improved precipitation data, which needs to be further verified. It has been reported that SPI with a short time scale is very sensitive to precipitation and is suitable for monitoring meteorological droughts, while SPI with medium and long time-scales (e.g., 6–12 months) are less sensitive to rainfall with obvious annual and decadal variations [42]. As a result, the SPI-3 was used to capture the occurrence of meteorological drought events in Hubei Province in this study. According to the record of meteorological disasters over Hubei province in the China Meteorological Disasters Yearbook, meteorological drought events that occurred in 2007 and 2013 were selected as representative cases to verify the applicability of the fused data in monitoring drought events.

**Figure 3:**
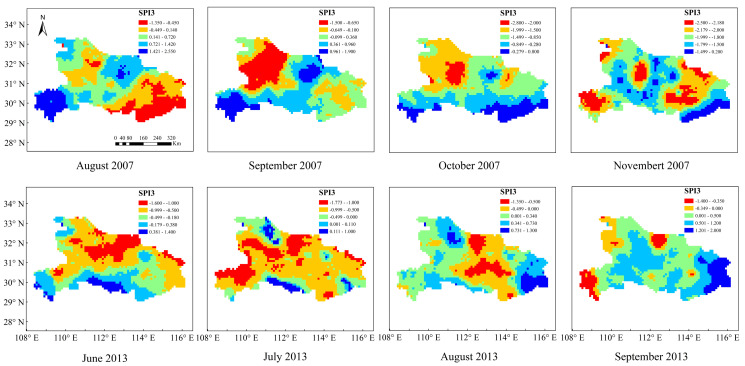
SPI-3 values based on the fused data in August, September, October, and November 2007 and June, July, August, and September 2013.

**Figure 4:**
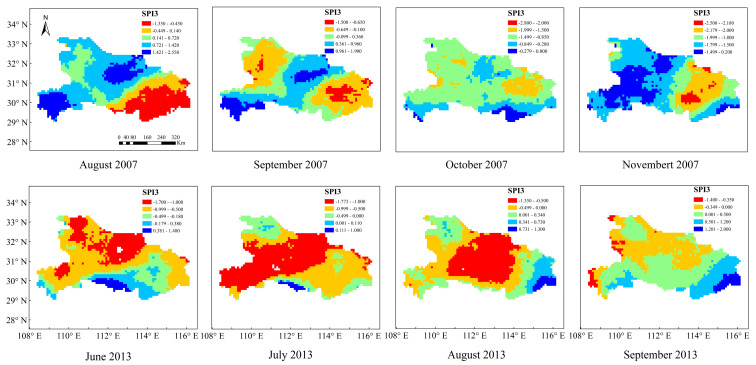
SPI-3 values based on the GPM data in August, September, October, and November 2007 and June, July, August, and September 2013.

It was recorded that obvious dry conditions occurred in Hubei Province from 25 April to 20 May, early June, early August, from 1 September to 25 October, and early November in 2007, especially for Shiyan, Xiaogan, Huanggang, and Wuhan City. Figure 3 and Figure 4 show the spatial distribution of the SPI-3 values calculated based on the GPM data and the fused data from August to November 2007 in Hubei province. In terms of capturing the onset and termination of drought events, SPI-3, based on the fused data, showed good agreement with records during this period. In August, the southeastern regions of Shiyan, Xiaogan, Huanggang, and Wuhan showed slight drought, while Shiyan exhibited moderate drought in September. In addition, the intensity of drought was increased in October, and became severe drought or extreme drought in November for most of the regions. However, Figure 4 plotted that SPI-3 based on the GPM data showed no drought or slight drought in Shiyan in September and October, which deviated from our records. Besides, there was a large error in capturing the onset and termination of drought. In general, fused data could capture the spatial extent of the drought event, as well as the drought intensity, more accurately than the GPM data.

In the summer of 2013, large-scale high-temperature weather from late July to mid-August, lasting 30–40 days without rainfall, occurred in Hubei Province. The concur-rent high temperature and rainfall deficit conditions caused severe droughts in 52 counties and cities, among which 30 counties and cities were extremely dry within Hubei province. The bottom panel of Figure 3 show that SPI-3 values detected drought in June and non-drought in September for most areas in Hubei province, indicating that it accurately captured the onset and termination of this historical drought event. In terms of the drought intensity of this event, most regions showed slight drought in June, and it reached the highest intensity of moderate drought or extreme drought in July. Subsequently, the drought decreased in the central part of Hubei Province and showed slight drought in August, and only a few areas showed slight drought in September. Compared with the SPI-3 based on the fused data, the bottom panel of Figure 4 plotted that SPI-3 based on the GPM data showed worse agreement with records. In September, SPI-3 still showed slight drought and moderate drought in most areas of Hubei province. The result showed that the improved GPM data presented a well-matched spatial pattern with the typical drought events. From 2002 to 2017, 8 drought events based on the fused data and 6 drought events based on the GPM data were accurately monitored among 10 meteorological droughts that occurred in Hubei province. Consequently, the SPI values calculated from the fused data can not only successfully monitor the occurrence and development of drought, but also accurately reflected the spatial distribution of drought intensity and outperformed the SPI values calculated from the original GPM data, which verified the applicability of the fused data for the following analysis of the spatio-temporal characteristics of drought over Hubei Province.

### 3.3. Spatio-Temporal Characteristics Analysis of Drought in Hubei Province

#### 3.3.1. Frequency Analysis and Spatio-Temporal Distribution of Drought with Different Severity

**Figure 5:**
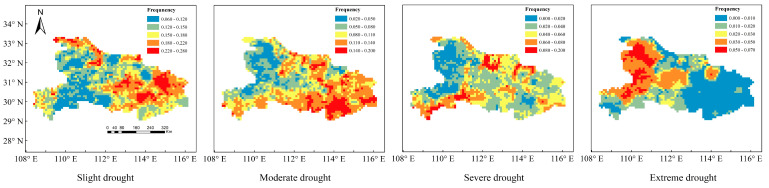
Distribution of drought frequency with different categories in Hubei Province.

The SPI-12 was calculated as the SPI series at a 12-month scale based on the fused data and was used to analyze the frequency of annual drought with different severity categories in Hubei province. Figure 5 plotted the spatial distribution of drought frequency with different categories, and it can be seen that the frequency of slight drought was the highest, followed by moderate drought, severe drought, and extreme drought in Hubei province. The central and eastern regions were prone to most of the droughts except for the extreme drought, while the western plateau region exhibited a low frequency of slight, moderate, and severe droughts, but, on the contrary, with a large frequency for the extreme drought. The results showed that the overall drought frequency in Hubei Province was 6~28% for slight droughts, 2~20% for moderate droughts, 0~20% for severe droughts, and 0~7% for extreme drought, respectively. In terms of the spatial distribution characteristics of different categories of drought, slight drought affected the whole province, mainly distributed in the north of Shiyan, Jingmen, and eastern area; moderate drought was concentrated in most areas of central and eastern Hubei and some areas of southwest China; the areas exhibiting severe drought were decreased, mainly distributed in the southwest and northern part of Hubei, and the distribution characteristics of extreme drought were significantly different from the previous droughts, which were mainly distributed in the western and northwestern regions. On the whole, drought with different categories showed apparent distribution characteristics in Hubei province, namely that slight, moderate, and severe drought mainly occurred in the central and eastern parts, while extreme drought mainly occurred in the northern and northwestern regions.

#### 3.3.2. Frequency Analysis and Spatial-Temporal Distribution of Seasonal Drought

**Figure 6:**
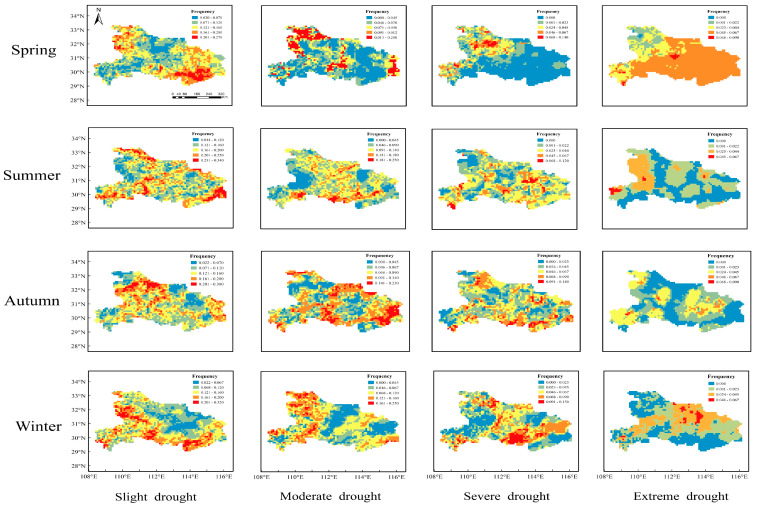
Seasonal drought frequency with different categories based on SPI-3.

**Figure 7:**
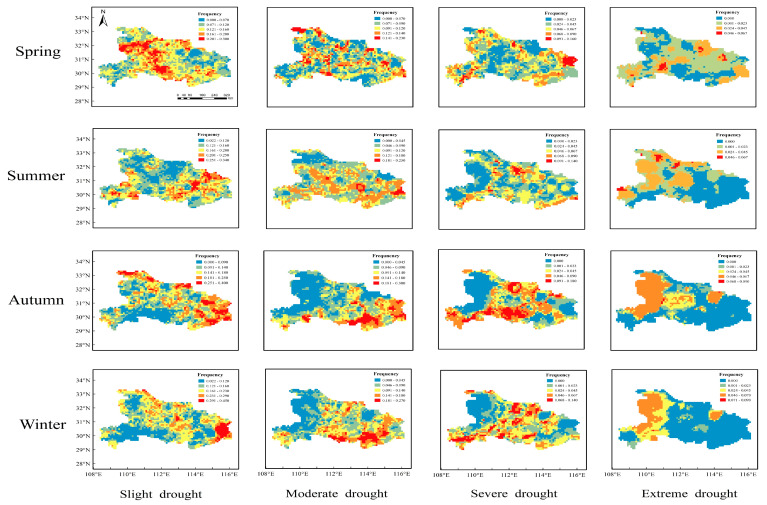
Seasonal drought frequency with different categories based on SPI-9.

It is necessary to study the seasonal distribution characteristics of droughts owing to their high incidence in Hubei Province, and the SPI at time scales of 3 months and 9 months were used to analyze short-term and medium-term drought characteristics, respectively. The following Figure 6 and Figure 7 plot the seasonal spatial distribution of droughts with different categories based on SPI-3 and SPI-9, respectively.

The results showed that the highest frequency of drought was most likely to occur in the summer in the northwest of Hubei Province, central Yichang, central Enshi, and southeast Huanggang. According to the distribution characteristics of droughts that occurred in different seasons, the average frequency of droughts was 27%, 33%, 31%, and 31% in spring, summer, autumn, and winter, respectively. In spring, the frequency of slight drought varied from 2% to 27%, mainly distributed in the northwest, Xianning, part of Jingzhou, and the southeast of Wuhan. The frequency of moderate and severe droughts was less than 20%, which were mainly distributed in the northwest and southwest regions, and there were few that occurred in the central region and Jianghan Plain. The extreme drought occurred only in the central region, and the maximum frequency was low at only 9%. In summer, drought affected more areas than spring drought, and it mainly occurred in the whole Hubei province with the category of slight drought, moderate drought, and severe drought, and the maximum frequency of slight drought was up to 34%. In autumn, the distribution characteristics of drought were similar to that in summer; conversely, the frequency of slight and moderate drought decreased with the maximum frequency being 30% and 23%, respectively, while the frequency of severe and extreme drought slightly increased. In winter, the distribution characteristics of drought were more distinct than other seasons since slight drought was mainly distributed in the west, southwest, and southeast; moderate drought was intensively concentrated in the western plateau, and severe and extreme drought was mainly distributed in the central part of Hubei province.

The results based on the SPI-9 showed that the largest frequency of droughts occurred in summer, with most droughts occurring in central and eastern regions and a few areas in western Hubei province. The slight drought exhibited the highest frequency among different drought categories. In spring, slight drought was mainly distributed in the western region and part of the central region, while moderate and severe drought occurred in many areas of the province. In summer, the highest frequency of slight drought was up to 34%, mainly distributed in the southwest and eastern regions. Moderate drought mainly covered Tianmen, Huanggang, and some other parts of the region, and the high frequency of severe drought was concentrated in the central and southern plains. In autumn, slight drought occurred in the eastern part of Hubei province and the northern part of Shiyan, with the frequency ranging from 25% to 40%. Moderate drought mainly occurred in the southwest part of Enshi and the central part of Hubei province. In winter, the distribution of drought was similar to autumn; however, the frequency of slight drought increased to 45%, while the frequency of moderate and severe drought decreased, and the affected area was narrowed.

In terms of the distribution characteristics of drought frequency based on the SPI-3 and SPI-9, we can conclude that drought based on the SPI-3 had a wide distribution coverage, with the highest frequency in summer and the lowest frequency in spring. All categories of droughts nearly affected the whole province. Distribution based on the SPI-9 was more regular, and the occurrence frequency was high in autumn and winter. Slight drought almost occurred in the whole province, moderate and severe drought mainly affected the plain area, and extreme drought mainly distributed in the western plateau area.

## 4. Conclusions

This study evaluated the applicability of TRMM, GPM, and CHIRPS satellite precipitation products based on the observation data of 26 meteorological stations in Hubei Province, then selected the products with the highest accuracy for data fusion. The spatial and temporal distribution characteristics of drought at different time scales and different drought categories from 2002 to 2017 over Hubei Province were analyzed based on the fused dataset. The main results are summarized as follows:

(a) The mean *CC* of GPM products was 0.91, which showed the best performance among the three satellite precipitation products, while the values for TRMM and CHIRPS were 0.88 and 0.85, respectively. Meanwhile, the mean *RMSE* value of GPM was lower than TRMM and CHIRPS, with 37.95, 41.68 and 45.85, respectively. In terms of *ME* and *BIAS* results and drought event monitoring, both TRMM and GPM performed well even though overestimates existed for precipitation in most areas, which was significantly better than the performance of CHIRPS.
(b) The GDA method was used for the fusion processing of GPM product data and measured data, then the SPI values were calculated from the fused data. Two typical meteorological drought events were selected in 2007 and 2013 to assess the performance of drought monitoring based on the fused data, and the result showed good agreement with the actual drought conditions. It was verified that the fused GPM product could monitor the onset, termination, intensity, and spatial distribution of drought events well.
(c) At an annual scale, the frequency of slight drought was the highest followed by moderate, severe, and extreme drought. Slight, moderate, and severe drought mainly occurred in the central and eastern parts, while extreme drought mainly occurred in the northern and northwestern regions. At the seasonal scale, the average drought frequency was the highest in summer and the lowest in spring; all categories of droughts nearly affected the whole province. Compared with the SPI-3, the SPI-9 was more stable, and the frequency of drought was higher in autumn and winter.

In summary, GPM had the best performance of drought monitoring and showed the best applicability in plain areas. The data fusion based on the GPM product and the observations took advantage of the precision and high spatial resolution in monitoring drought events. The frequency of slight drought was the highest annually and mainly distributed in central and eastern provinces. Among the seasonal droughts, summer drought was the most frequent, short-term drought monitored by the SPI-3 affected the whole province, and medium drought monitored by the SPI-9 mainly affected the plain area. The accuracy assessment of precipitation products, data fusion processing, and spatio-temporal analysis of drought in Hubei province is expected to provide a reference for relevant research in other regions.

## Figures and Tables

**Table 1:** Basic information of meteorological stations in Hubei Province.

Station	Lon (°E)	Lat (°N)	Name	Station	Lon (°E)	Lat (°N)	Name
57,251	110.21	32.59	Yunxi	57,445	109.38	30.36	Jianshi
57,259	110.46	32.02	Fangxian	57,447	109.22	30.16	Exi
57,265	111.40	32.25	Laohekou	57,458	110.37	30.10	Wufeng
57,278	111.57	32.05	Xiangyang	57,461	111.05	30.42	Yichang
57,279	112.40	32.09	Zaoyang	57,476	112.05	30.24	Jinzhou
57,355	110.24	31.04	Badong	57,482	113.51	30.56	Xiaogan
57,358	110.36	31.00	Zigui	57,483	113.19	30.39	Tianmen
57,359	110.46	31.14	Xingshan	57,493	114.20	30.24	Wuchang
57,363	111.47	31.47	Nanzhang	57,494	114.17	30.38	Wuhan
57,378	112.34	31.10	Zhongxiang	57,545	109.29	29.34	Laifeng
57,381	113.2	31.43	Suizhou	57,595	114.23	29.36	Tongshan
57,395	114.04	31.34	Dawu	58,402	115.37	30.44	Yingshan
57,399	114.58	31.11	Macheng	58,407	115.01	30.15	Huangshi

Note: Lon indicates for the longitude and Lat indicates for the latitude.

**Table 2:** Drought category classification based on SPI.

Number	Drought Categories	SPI
D_0_	No drought	SPI ≥ −0.5
D_1_	Slight drought	−0.5 < SPI ≤ 1.0
D_2_	Moderate drought	−1.5 < SPI ≤ −1.0
D_3_	Severe drought	−2.0 < SPI ≤ −1.5
D_4_	Extreme drought	SPI ≤ −2.0

**Table 3:** The *POD* and *FAR* values of TRMM, CHIRPS, and GPM at 0.5 mm/d and 5 mm/d precipitation thresholds based on 26 meteorological stations.

Station	0.5 mm/d						5 mm/d					
	TRMM		GPM		CHIRPS		TRMM		GPM		CHIRPS	
	*POD*	*FAR*	*POD*	*FAR*	*POD*	*FAR*	*POD*	*FAR*	*POD*	*FAR*	*POD*	*FAR*
57,251	0.96	0.09	0.97	0.06	0.97	0.09	0.55	0.37	0.73	0.16	0.64	0.44
57,259	0.97	0.08	0.99	0.05	0.99	0.09	0.80	0.48	0.80	0.36	0.85	0.48
57,265	0.93	0.01	1.00	0.04	1.00	0.10	0.88	0.29	0.82	0.22	0.82	0.42
57,278	0.93	0.01	1.00	0.05	1.00	0.10	0.88	0.29	0.72	0.43	0.82	0.42
57,279	0.94	0.07	1.00	0.07	1.00	0.11	0.82	0.33	0.88	0.40	0.65	0.35
57,355	0.99	0.07	0.99	0.04	0.99	0.09	0.98	0.38	0.93	0.16	0.63	0.24
57,358	0.94	0.02	0.97	0.03	0.97	0.07	0.72	0.32	0.77	0.23	0.49	0.30
57,359	0.94	0.04	0.99	0.04	0.99	0.11	0.80	0.50	0.90	0.39	0.43	0.35
57,363	0.94	0.07	1.00	0.08	1.00	0.12	0.76	0.53	0.64	0.45	0.80	0.52
57,378	0.94	0.06	1.00	0.08	1.00	0.10	0.79	0.28	0.86	0.32	0.76	0.27
57,381	0.93	0.04	1.00	0.04	0.97	0.12	0.81	0.21	0.81	0.21	0.88	0.28
57,395	0.96	0.05	1.00	0.08	1.00	0.13	0.86	0.31	0.86	0.38	0.86	0.44
57,399	0.97	0.04	1.00	0.07	1.00	0.09	0.72	0.41	0.83	0.39	0.79	0.38
57,445	0.94	0.02	1.00	0.03	1.00	0.06	0.79	0.21	0.83	0.16	0.90	0.24
57,447	0.92	0.00	1.00	0.00	1.00	0.02	0.82	0.14	0.82	0.06	0.88	0.20
57,458	0.94	0.02	1.00	0.03	1.00	0.04	0.91	0.34	0.91	0.31	0.85	0.35
57,461	0.95	0.04	1.00	0.07	1.00	0.09	0.95	0.42	0.88	0.39	0.88	0.33
57,476	0.94	0.02	1.00	0.07	1.00	0.06	0.74	0.29	0.88	0.30	0.82	0.38
57,482	0.92	0.07	1.00	0.07	1.00	0.08	0.74	0.35	0.89	0.35	0.80	0.39
57,483	0.93	0.05	1.00	0.06	1.00	0.07	0.80	0.31	0.93	0.43	0.77	0.45
57,493	0.97	0.02	1.00	0.03	1.00	0.05	0.88	0.28	0.85	0.31	0.81	0.34
57,494	0.96	0.03	1.00	0.04	1.00	0.06	0.95	0.28	0.93	0.31	0.83	0.40
57,545	0.93	0.01	1.00	0.01	1.00	0.05	0.90	0.25	0.88	0.21	0.86	0.30
57,595	0.99	0.02	1.00	0.02	1.00	0.04	0.85	0.21	0.85	0.08	0.88	0.26
58,402	0.97	0.07	1.00	0.06	1.00	0.08	0.91	0.38	0.85	0.38	0.89	0.38
58,407	0.95	0.01	1.00	0.03	1.00	0.04	0.87	0.30	0.91	0.28	0.89	0.34
Average	0.947	0.040	0.996	0.048	0.997	0.079	0.827	0.325	0.844	0.295	0.787	0.356

## Data Availability

Not applicable.
